# Evaluation of L-Alanine Metabolism in Bacteria and Whole-Body Distribution with Bacterial Infection Model Mice

**DOI:** 10.3390/ijms24054775

**Published:** 2023-03-01

**Authors:** Yuka Muranaka, Miki Matsue, Asuka Mizutani, Masato Kobayashi, Kakeru Sato, Ami Kondo, Yuri Nishiyama, Shusei Ohata, Kodai Nishi, Kana Yamazaki, Ryuichi Nishii, Naoto Shikano, Shigefumi Okamoto, Keiichi Kawai

**Affiliations:** 1 Division of Health Sciences, Graduate School of Medical Sciences, Kanazawa University, 5-11-80 Kodatsuno, Kanazawa 920-0942, Japan; 2 Ishikawa Prefectural Institute of Public Health and Environmental Science, 1-11 Taiyogaoka, Kanazawa 920-1154, Japan; 3 Faculty of Health Sciences, Institute of Medical, Pharmaceutical and Health Sciences, Kanazawa University, 5-11-80 Kodatsuno, Kanazawa 920-0942, Japan; 4 Department of Radiation Technology, Toyama Red Cross Hospital, 2-1-58 Ushijimahonmachi, Toyama 930-8562, Japan; 5 Department of Radioisotope Medicine, Atomic Bomb Disease Institute, Nagasaki University, 1-12-4 Sakamoto, Nagasaki 852-8523, Japan; 6 Department of Molecular Imaging and Theranostics, Institute for Quantum Medical Science, Quantum Life and Medical Science Directorate, National Institutes for Quantum Science and Technology, 4-9-1 Anagawa, Inage, Chiba 263-8555, Japan; 7 Department of Radiological Sciences, Ibaraki Prefectural University of Health Sciences, 4669-2 Ami, Inashiki 300-0394, Japan; 8 Advanced Health Care Science Research Unit, Innovative Integrated Bio-Research Core Institute for Frontier Science Initiative, Kanazawa University, 5-11-80 Kodatsuno, Kanazawa 920-0942, Japan; 9 Biomedical Imaging Research Center, University of Fukui, 23-3 Matsuokashimoaizuki, Eiheiji, Fukui 910-1193, Japan

**Keywords:** amino acids, alanine, bacterial imaging, nuclear medicine imaging, bacterial infection

## Abstract

The World Health Organization has cautioned that antimicrobial resistance (AMR) will be responsible for an estimated 10 million deaths annually by 2050. To facilitate prompt and accurate diagnosis and treatment of infectious disease, we investigated the potential of amino acids for use as indicators of bacterial growth activity by clarifying which amino acids are taken up by bacteria during the various growth phases. In addition, we examined the amino acid transport mechanisms that are employed by bacteria based on the accumulation of labeled amino acids, Na^+^ dependence, and inhibitory effects using a specific inhibitor of system A. We found that ^3^H-L-Ala accurately reflects the proliferative activity of *Escherichia coli* K-12 and pathogenic EC-14 in vitro. This accumulation in *E. coli* could be attributed to the amino acid transport systems being different from those found in human tumor cells. Moreover, biological distribution assessed in infection model mice with EC-14 using ^3^H-L-Ala showed that the ratio of ^3^H-L-Ala accumulated in infected muscle to that in control muscle was 1.20. By detecting the growth activity of bacteria in the body that occurs during the early stages of infection by nuclear imaging, such detection methods may result in expeditious diagnostic treatments for infectious diseases.

## 1. Introduction

Antimicrobial resistance (AMR) poses significant risks to people around the world, with approximately 33,000 deaths annually attributed to AMR in Europe [1]. The World Health Organization cautions that if this trend persists, AMR will be responsible for an estimated 10 million deaths per year by 2050 [2,3].

The quandary of AMR has become a pressing matter that must be addressed. Consequently, in order to facilitate prompt and accurate diagnosis and treatment of infectious diseases, a diagnostic methodology capable of detecting pathogenic bacteria at the locus of infection in the early stages of infection is essential and urgent.

Nuclear medicine imaging has attracted considerable attention as a novel means of examining bacterial infections. This methodology enables non-invasive examination and diagnosis through imaging the state of the patient’s body with radiopharmaceutical agents that are administered to the patient [4]. In this study, we posited that if the proliferation of bacteria that occurs in the body during the early stages of infection can be captured by imaging, then such detection methods may result in new and expeditious diagnostic treatments for infectious diseases.

Bacterial growth can be classified into the lag phase, log phase, stationary phase, and death phase [5]. Given that bacteria exhibit varying degrees of proliferative activity during each phase, we surmised that the prompt detection of changes in proliferative activity may facilitate the implementation of appropriate treatments. Since the glucose-based diagnostic imaging agent 2-deoxy-2-[^18^F]fluoro-D-glucose (^18^F-FDG), which is utilized primarily in clinical practice [6,7,8], can be taken up by both bacterial pathogens and inflammatory cells associated with bacterial infection, ^18^F-FDG has been investigated as a potential diagnostic imaging agent for acute infectious diseases [9,10,11]. However, despite the reported ability of ^18^F-FDG to clarify the presence or absence of bacterial infection, limited research has been conducted on the ability of this agent to detect the growth activity of pathogenic bacteria in detail. As a method aimed at the specific detection of bacterial infections, ^99m^Tc-based imaging agent, such as ^99m^Tc ciprofloxacin [12] were investigated.

Accordingly, this study focused on the potential of amino acid-based diagnostic imaging agents for use as novel diagnostic imaging agents that utilize bacterial growth activity as an indicator of infection. Amino acids are necessary for bacterial growth [13], and it is well-established that the transport function of amino acids is ubiquitous in bacteria, which can take up many types of amino acids.

Furthermore, our laboratory has developed a method to evaluate the contribution of amino acid transport systems in tumor cells for the development of amino acid tumor imaging agents targeting human tumor cells in previous study. We have evaluated the contribution of amino acid transporters, especially system A and system L, which are known to be highly expressed in tumor cells [14,15,16]. The application of this method to bacteria is important for the development of imaging agents for identifying amino acid pathogens and for the differential diagnosis of bacterial infections. 

In this study, we investigated the utility of amino acids as indicators of bacterial growth activity by conducting a detailed examination of which amino acids are taken up by bacteria during in the lag phase, log phase, stationary phase, and death phases of bacterial growth. In addition, we examined the mechanisms involved in amino acid transport in bacteria based on the Na^+^ dependence and inhibitory effects using a specific inhibitor of system A.

## 2. Results

### 2.1. Accumulation of ^3^H-L-Ala, ^3^H-L-Met, ^3^H-L-Glu, and ^3^H-L-His in E. coli K-12

**Figure 1:**
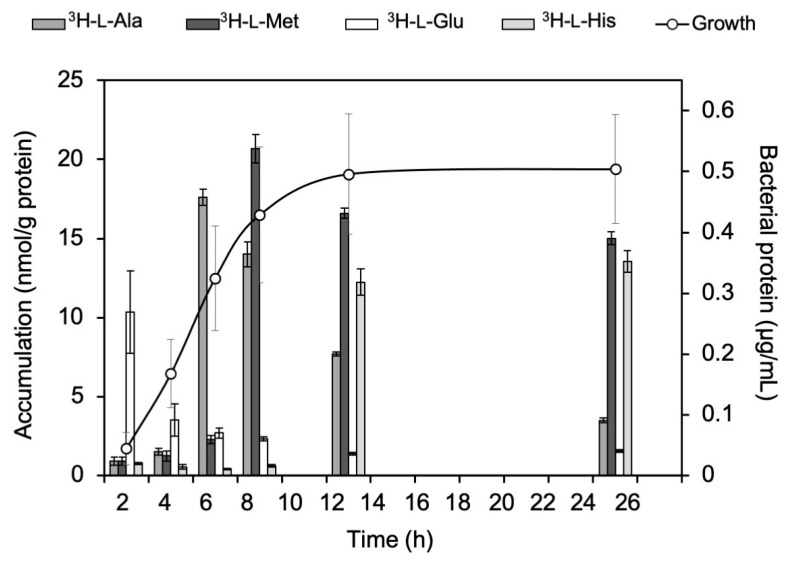
Accumulation of ^3^H-L-Ala, ^3^H-L-Met, ^3^H-L-Glu, and ^3^H-L-His in *E. coli* K-12 at each growth stage. At 2 h, ^3^H-L-Glu showed the highest accumulation. At 6 h and 8 h, ^3^H-L-Ala and ^3^H-L-Met accumulated more than the other amino acids. At 12 h and 24 h, the accumulation of ^3^H-L-Met and ^3^H-L-His was higher than that of the other amino acids.

The accumulation of ^3^H-L-Ala, ^3^H-L-Met, ^3^H-L-Glu, and ^3^H-L-His in *E. coli* K-12 in each growth stage is shown in Figure 1. The findings showed that the lag phase of K-12 (2 h) had the highest accumulation of ^3^H-L-Glu. In the log phase (6 h and 8 h), higher levels of ^3^H-L-Ala and ^3^H-L-Met were accumulated compared to the other amino acids. In the stationary phase (12 h and 24 h), the accumulation of ^3^H-L-Met and ^3^H-L-His was the highest among the amino acids.

### 2.2. Accumulation of ^3^H-L-Ala, ^3^H-L-Met, ^3^H-L-Glu, and ^3^H-L-His in E. coli EC-14

**Figure 2:**
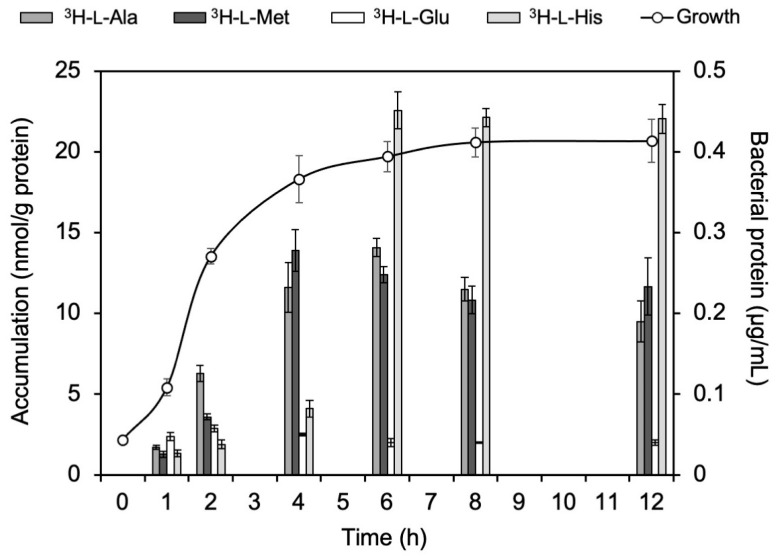
Accumulation of ^3^H-L-Ala, ^3^H-L-Met, ^3^H-L-Glu, and ^3^H-L-His in *E. coli* EC-14 at each growth stage. At 1 h, the accumulation of most of the amino acids was low. At 2 h and 4 h, ^3^H-L-Ala and ^3^H-L-Met accumulated more than the other amino acids. At 6, 8, and 12 h, ^3^H-L-His showed the highest accumulation levels.

The accumulation of ^3^H-L-Ala, ^3^H-L-Met, ^3^H-L-Glu, and ^3^H-L-His in *E. coli* EC-14 at each growth stage is shown in Figure 2. First, in the lag phase of EC-14 (1 h), the accumulation of most amino acids was low. In the log phase (2 and 4 h), ^3^H-L-Ala and ^3^H-L-Met showed the highest accumulation among the amino acids. In the stationary phase (6, 8, and 12 h), ^3^H-L-His showed the highest accumulation.

### 2.3. Comparing the Accumulation of ^3^H-L-Ala in E. coli K-12 and EC-14

**Figure 3:**
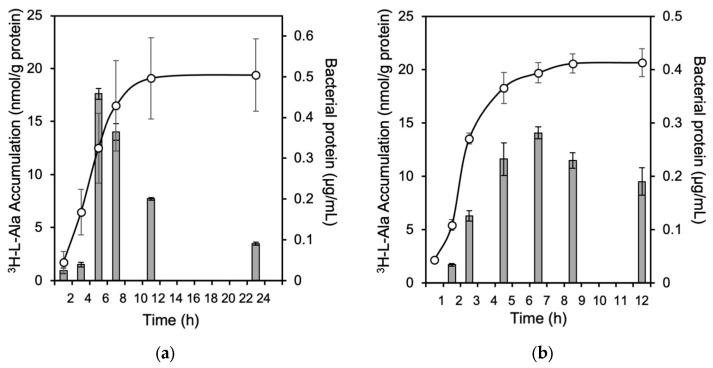
Accumulation of ^3^H-L-Ala in (**a**) *E. coli* K-12 and (**b**) EC-14 at each growth stage. H-L-Ala accumulation in K-12 showed the highest uptake during the log phase, but not much uptake during the stationary phase. In contrast, the accumulation by EC-14 remained high from the logarithmic growth stage to the stationary phase.

The accumulation of ^3^H-L-Ala in *E. coli* K-12 and EC-14 at each growth stage is shown in Figure 3. ^3^H-L-Ala accumulation in K-12 showed the highest accumulation during the log phase, but not much accumulation during the stationary phase. Conversely, the accumulation of EC-14 remained high from the log phase to the stationary phase. 

### 2.4. Inhibition of Amino Acid Accumulation in Bacteria and Cancer Cells

**Figure 4:**
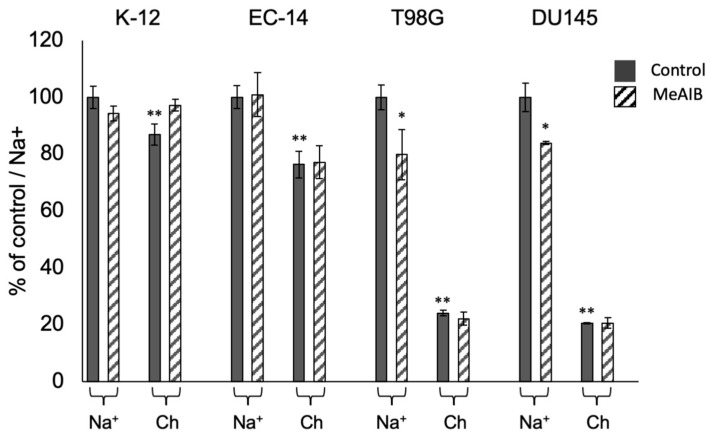
Accumulation of L-Ala in *E. coli* K-12 and EC-14. Accumulation was compared with that in human tumor cells (i.e., DU145 human prostate cancer cells and T98G human glioblastoma cells) under control and specific inhibitor of system A, MeAIB-treated conditions. For L-Ala, comparing the accumulation of labeled amino acids in Ch-PBS (Na^+^-free medium) and the accumulation of labeled amino acids in Na^+^-PBS, 13% of K-12 and 24% of EC-14 were Na^+^-dependent. Moreover, comparing the inhibitory effect of MeAIB on L-Ala accumulation, K-12 and EC-14 showed no significant inhibitory effect on L-Ala accumulation by MeAIB, which is observed in tumor cells. * *p* < 0.05, ** *p* < 0.01 vs. control/Na+.

To determine some characteristics of the amino acid transport system in bacteria, we compared it with the transport system in human cancer cells. The accumulation of L-Ala in *E. coli* K-12 and EC-14 was compared to that in human tumor cells, i.e., human prostate cancer cell line DU145 and human glioblastoma cell line T98G, under control and specific inhibitor-treated conditions (i.e., treatment with MeAIB). In L-Ala, 13% of K-12 and 24% of EC-14 were Na^+^-dependent, but the percentage was small compared to the more than 75% Na^+^-dependence of tumor cells. Compared to the inhibitory effect of MeAIB on L-Ala accumulation, the K-12 and EC-14 cells showed no significant inhibitory effect on L-Ala accumulation by MeAIB, which is observed in tumor cells (DU145 and T98G) (Figure 4).

### 2.5. Biological Distribution of E. coli EC-14 Infection Model Mice Using ^3^H-L-Ala

Table 1 shows the biological distribution of ^3^H-L-Ala in EC-14 infection model mice. ^3^H-L-Ala showed a maximum contrast of 1.2 at 2 h after infection, 60 min after ^3^H-L-Ala injection. This contrast is defined as the ratio of accumulation by weight of infected and uninfected muscle. The ratios of infected muscle to blood were 0.79 and 0.75 at 2 and 8 h after infection, respectively, and 0.89 and 1.31 at 15 and 60 min after ^3^H-L-Ala injection, respectively.

## 3. Discussion

In this study, we investigated the utility of amino acids as indicators of bacterial growth and activity by conducting a detailed examination of which amino acids are accumulated by bacteria during the lag phase, log phase, stationary phase, and death phases of growth. We then clarified the amino acid transport properties in bacteria by investigating the accumulation of labeled amino acids, Na^+^ dependence, and inhibitory effects using MeAIB, specific inhibitor of system A. We previously focused on MeAIB in our research group for application to positron emission tomography imaging in cancer [17].

First, in the in vitro bacterial accumulation study, the accumulation of neutral amino acids, ^3^H-L-Ala and ^3^H-L-Met, increased from the lag phase to the log phase (Figure 1 and Figure 2). This indicates that neutral amino acids are useful as indicators of proliferative activity in K-12 and EC-14. The accumulation of the acid amino acid ^3^H-L-Glu in EC-14, unlike that in K-12, did not change during each growth phase and remained constant (Figure 1 and Figure 2). One of the reasons for this disparity in the accumulation characteristics of K-12 and EC-14 may be due to differences in amino acid transporters. It has been reported that K-12 has both ATP-binding cassette transporters (ABC transporters) and ion-driven transporters for L-glutamic acid and L-lysine, while the other natural amino acids used in our experiments have only one of the two transporters [18]. On the other hand, the details of the amino acid transporters of EC-14 are unknown because few reports have been published to date, but it is possible that L-glutamic acid and L-lysine have only one of the two transporters, i.e., the ABC transporter or an ion-driven transporter, and this difference may be related to the difference in uptake between the two strains.

Furthermore, ^3^H-L-Ala showed the largest accumulation among the neutral amino acids in both K-12 and EC-14 (Figure 1 and Figure 2). Therefore, it is possible that ^3^H-L-Ala may best reflect the proliferative activity of *E. coli*. One of the reasons for the high accumulation of ^3^H-L-Ala during the log phase may be due to cell wall synthesis. Peptidoglycan, one of the components of the cell wall, is composed of a sugar chain consisting of N-acetylglucosamine (GlcNAc) and N-acetylmuramic acid (MurNAc) linked alternately by β-1.4 bonds, as well as L-Ala, D-Glu, L-diaminopimelic acid (L-DAP), and D-Ala [19]. The highest accumulation of ^3^H-L-Ala during the log phase is considered to be due to its incorporation into the cell wall as a component of peptidoglycan, as the synthesis of the cell wall is promoted by the activation of proliferation [20].

^3^H-L-Ala is taken up by the bacteria as they grow. In pathogenic EC-14, ^3^H-L-Ala accumulation is maintained even during the stationary phase, suggesting that the take up of ^3^H-L-Ala may be maintained as the pathogenic bacteria proliferate. Based on the assumption of the existence of a specific transport system for the accumulation of alanine, we performed the accumulation inhibition experiments. In this study, the amino acid transport system and inhibitors were determined based on studies performed in human cancer cells [16]. Previously, we developed a method to evaluate the contribution of amino acid transport systems in tumor cells for the development of amino acid tumor imaging agents targeting human tumor cells [14,15,16]. By using this evaluate method, we investigated the accumulation inhibition experiments. Moreover, amino acid transport systems in humans can be divided into two major types: Na^+^-dependent transport systems, which show transport activity in the presence of Na^+^, and Na^+^-independent transport systems, which show transport activity regardless of the presence or absence of Na^+^. We aimed to elucidate these transport properties in bacteria as well. The results showed that for ^3^H-L-Ala, 13% of K-12 and 24% of EC-14 were Na^+^-dependent, but the percentage was small compared to the Na^+^-dependence levels of more than 75% observed in tumor cells (Figure 4). Comparing the inhibitory effect of MeAIB on ^3^H-L-Ala accumulation, the K-12 and EC-14 showed no significant inhibitory effect on ^14^C-L-Ala accumulation by MeAIB, which is observed in tumor cells. The findings revealed that the amino acid transport system employed by *E. coli* differs from that found in human tumor cells. The application of this method which evaluate the contribution of amino acid transporters for bacteria is important for the development of imaging agents for identifying pathogens.

Table 1 shows the biological distribution of ^3^H-L-Ala in EC-14 infection model mice. In vivo, ^3^H-L-Ala had a maximum contrast of 1.20 at 2 h after infection. This contrast is defined as the ratio of amino acid accumulation by weight of infected and uninfected muscle. The low accumulation of radiotracer in the heart may be better suited than ^18^F-FDG, which showed high levels of physiological accumulation [21], for detecting infections in the upper body and other parts of the body. Also in the biological distribution, the detection window of infection was narrow because the maximum contrast of 1.2 at 2 h after infection was close to 1.0 (showing no contrast). We have to pay attention to it raised a concern that there could be false negative results in practice. A limitation of this study was that we could not perform imaging as our facility is not equipped with a cyclotron or an automatic synthesizer for ^11^C labeling. However, the results of the whole-body biodistribution are considered to be equivalent to those of whole-body imaging [22].

## 4. Materials and Methods

[2,3-^3^H]-L-Ala (^3^H-L-Ala), [S-methyl-^3^H]-L-Met (^3^H-L-Met), [2,3,4-^3^H]-L-glutamic acid (^3^H-L-Glu), and [ring 2,5-^3^H]-L-histidine (^3^H-L-His) (American Radiolabeled Chemicals; St. Louis, MO, USA) were used in this study. Only in the cancer cell accumulation inhibition experiment study, was [^14^C(U)]-L-alanine (^14^C-L-Ala) used (PerkinElmer, Waltham, MA, USA). 

### 4.1. Bacterial Strain and Culture Conditions

The bacterial strains used in this study were *Escherichia coli* K-12 (DH5α strain) and *E. coli* EC-14 (Shionogi, Osaka, Japan), which was used as the clinical isolate strain [23]. In the pre-cultivation process, 1:100 mixtures of EC-14 stock solutions containing 50% glycerol and THY medium made up of Todd–Hewitt Broth (Becton, Dickinson and Company, Franklin Lakes, NJ, USA) and 0.2% yeast extract (Becton, Dickinson and Company) were used. The mixtures were shaken at 37 °C for 12–14 h before EC-14 was seeded in amino acid-free Dulbecco’s Modified Eagle’s Medium (DMEM; FUJIFILM Wako Pure Chemical Corporation, Osaka, Japan) and incubated with shaking at 37 °C. A Pierce™ BCA Protein Assay Kit (Thermo Fisher Scientific, Waltham, MA, USA) was used to measure bacterial protein concentrations.

### 4.2. Human Tumor Cell Line and Culture Conditions

For brain tumor cells, human glioblastoma cell line T98G was used. For prostate cancer cells, the DU145 cell line was used. These cell lines were purchased from American Type Culture Collection (Manassas, VA, USA) and have been used in previous studies by our research group [14,15].

First, human tumor cells were cultured in a cell culture dish. The cells were washed twice with phosphate buffered saline (PBS; pH 7.3) (Medical & Biological Laboratories Co., Ltd., Aichi, Japan) and then collected with 0.25% trypsin-EDTA solution. The reaction was then stopped in culture medium, and the cells were seeded in 24-well multi-well plates at 1 × 10^5^ cells/well. The cells were incubated for approximately 24 h after seeding. DMEM containing 10% fatal bovine serum (FBS) and 1% sodium pyruvate was used for culturing T98G cells. RPMI 1640 medium containing 10% FBS and 1% sodium pyruvate was used for culturing DU145 cells.

### 4.3. Accumulation of ^3^H-L-Ala, ^3^H-L-Met, ^3^H-L-Glu, and ^3^H-L-His in E. coli K-12 and EC-14

*E. coli* K-12 at 1.3 × 10^6^ CFU/200 µL and EC-14 at 1.2 × 10^8^ CFU/100 µL were seeded in 5 mL of amino acid-free DMEM and incubated for 1, 2, 4, 6, 8, 12, and 24 h. After incubation, 37 kBq/10 µL of ^3^H-L-Ala, ^3^H-L-Met, ^3^H-L-Glu, and ^3^H-L-His were added to the bacterial solution and incubated for 5 min at 37 °C with gentle shaking. K-12 and EC-14 were collected by centrifugation at 7000× *g* for 10 min at 4 °C, washed three times with 5 mL of PBS.

K-12 and EC-14 were lysed by the addition of 1 mL of 0.1 M NaOH. The radioactivity of the mixture made up of 500 µL of bacterial lysate and 5 mL of liquid scintillation cocktail (ULTIMA GOLD, Perkin Elmer, Waltham, MA, USA) was measured using a liquid scintillation counter (LSC-5100; Hitachi Aloka Medical, Tokyo, Japan).

### 4.4. Inhibition of Amino Acid Accumulation in Bacteria

In this study, we applied the experimental methods used to study human cancer cells to bacteria. *E. coli* K-12 and EC-14 were cultured under the same conditions as described in Section 4.3. The pre-cultured bacteria solution was incubated at 37 °C in 20 mL of amino acid-free DMEM at 160 rpm. The relationship between the number of bacteria and incubation time was expressed as a growth curve for *E. coli* to show the lag phase (K-12: 2 h, EC-14: 1 h), the log phase (K-12: 6 h, EC-14: 3 h), the stationary phase (K-12: 12 h, EC-14: 6 h), and the death phase. The lag phase of bacteria is the early phase of the growth period, and the log phase means the period when the bacteria proliferate actively due to active enzyme activity in the bacteria. And the death phase means the period when the ratio of cell growth to cell death is equal due to nutrient deprivation and accumulation of toxic metabolites.

In this experiment, after incubation of K-12 for 6 h and EC-14 for 3 h, which is the log phase, the DMEM culture medium was replaced with medium containing 137 mM NaCl, 2.7 mM KCl, 8 mM Na_2_HPO_4_, 1.5 mM KH_2_PO_4_, 5.6 mM D-glucose, 0.9 mM CaCl_2_, and 0.5 mM MgCl_2_ Na^+^-PBS (pH 7.3), or choline-PBS (Ch-PBS; pH 7.3), with NaCl and Na_2_HPO_4_ in Na^+^-PBS replaced with the same concentrations of chlorine-Cl and K_2_HPO_4_, respectively. Ch-PBS is the Na^+^ -free medium used for contrast with Na^+^ contain medium. Following solvent exchange, 7.4 kBq of labeled amino acids and α-(methylamino)isobutyric acid (MeAIB; Sigma-Aldrich, St. Louis, MO, USA), which is a specific inhibitor of system A, were added to a final concentration of 1.0 mM and uptake of the mixture was promoted by incubating in a water bath at 37 °C for 5 min with gentle shaking. After centrifugation at 7000× *g* for 6 min at 4 °C, the supernatant was removed, and the pellet was loosened, 1.0 mL of Na^+^-PBS and Ch-PBS were added, and centrifugation was performed twice to wash the pellet.

After washing, the supernatant was removed, the pellet was loosened, and 1.0 mL of 0.1 M NaOH was added to lyse the *E. coli* to prepare the sample lysate. A 500 µL aliquot was then removed from the sample lysate and the radioactivity was measured using a liquid scintillation counter. A Pierce™ BCA Protein Assay Kit was used to measure the bacterial protein.

To calculate the contribution of amino acid transporter systems, we used the methods reported by Kobayashi et al. [16]. The accumulation of the labeled amino acids under each condition was thus expressed as a relative value (percentage of control/Na^+^ [%]) based on the assumption that the accumulation in Na^+^-PBS without inhibitor (Na^+^-control) was 100%. Moreover, to calculate the Na^+^ dependency, the accumulation of labeled amino acids in Ch-PBS (Na^+^-free medium) was expressed as a relative value based on the accumulation of the control, in which the accumulation of labeled amino acids in Na^+^-PBS was 100%. In this study, Ch-PBS is the Na^+^-free medium used for contrast with Na^+^-containing medium.

### 4.5. Inhibition of Amino Acid Accumulation in Cancer Cells

For competitive inhibition experiments, the culture medium in the wells was removed and the cells were incubated in 300 μL of Na^+^-PBS (pH 7.3) buffer containing 137 mM NaCl, 2.7 mM KCl, 8 mM Na_2_HPO_4_, 1.5 mM KH_2_PO_4_, 5.6 mM D-glucose, 0.9 mM CaCl_2_, and 0.5 mM MgCl_2_ or Na^+^-choline-PBS (pH 7.3), in which NaCl and Na_2_HPO_4_ in PBS were replaced with the same concentrations of choline-Cl and K_2_HPO_4_, and the plates were preincubated at 37 °C for 10 min.

After 10 min, 9.25 kBq/well of ^14^C-L-Ala and 1 mM of MeAIB were added and the plates were incubated at 37 °C. After incubation, the solution in the wells was removed, and the cell surface and wells were briefly washed twice with ice-cold Na^+^-PBS or Ch-PBS. Then, 500 μL of 0.1 M NaOH was added to each well to lyse the cells, 350 μL of the cell lysate was mixed with liquid scintillation cocktail, and radioactivity was measured with a liquid scintillation counter. The remaining cell lysate was used to measure the cell protein mass in each well and to correct for the accumulation count of labeled amino acids in each well. These results were used to evaluate the contribution of the inhibitor.

### 4.6. Biological Distribution of E. coli EC-14 Infection Model Mice Using ^3^H-L-Ala

All procedures of animal study were approved by the Animal Care Committee of Kanazawa University (AP-183983) and were conducted in accordance with the international standards for animal welfare and institutional guidelines. 

EC-14 (approximately 5 × 10^6^ CFU/100 µL) were injected into the muscle of the hind leg of mice (n = 4), described in Muranaka et al. [21]. At 2 and 8 h after infection, mice were administered 50 kBq/50 µL of ^3^H-L-Ala intravenously. Prior to administration, the mice were fasted for 4 h. Mice were then euthanized at 15 min (n = 4) and at 60 min (n = 4) after administration, and approximately 100 mg of blood, heart, lung, liver, kidney, spleen, infected perineal muscle, and non-infected perineal muscle (control muscle) tissues were collected.

The collected organs were divided into approximately 100 mg portions and dissolved by adding 1.0 mL of solubilizer (Solvable, PerkinElmer, Waltham, MA, USA) and crushed using a disposable homogenizer (BioMasher^®^, Nippi Inc., Tokyo, Japan). The radioactivity of ^3^H-L-Ala was measured using a liquid scintillation counter (LSC-5100, Hitachi Aloka Medical). 

### 4.7. Statistical Analysis 

Data are reported as means and standard deviation of percent injected dose per gram of tissue (%ID/g) and analyzed using the F-test and Student’s *t*-test. A *p* value of less than 0.01 or 0.05 was considered indicative of a statistically significant difference. 

## 5. Conclusions

^3^H-L-Ala reflects accurately the proliferative activity of *E. coli* K-12 and EC-14. This utility of amino acids as indicators of bacterial growth activity may result in expeditious diagnostic treatments for infectious diseases. In addition, this accumulation in *E. coli* could be attributed to the amino acid transport systems being different from those found in human tumor cells.

## Figures and Tables

**Table 1:** Biological distribution of ^3^H-L-Ala in EC-14 infection model mice.

Time after ^3^H-L-Ala Injection	15 min	60 min	15 min	60 min
Organ (%ID/g)	2 h after Infection		8 h after Infection	
Blood	6.54 ± 0.57	5.64 ± 0.35	5.10 ± 0.55	3.05 ± 0.14
Heart	4.33 ± 0.44	3.87 ± 0.37	3.90 ± 0.24	3.65 ± 0.26
Lung	7.66 ± 1.57	5.60 ± 1.31	6.17 ± 0.67	5.22 ± 0.30
Liver	4.26 ± 0.70	4.88 ± 1.03	3.96 ± 0.65	4.23 ± 0.54
Spleen	17.11 ± 2.39	15.64 ± 4.53	9.94 ± 0.73	7.60 ± 1.22
Kidney	8.90 ± 0.67	7.78 ± 0.54	7.06 ± 1.10	6.76 ± 0.74
Infected muscle	5.14 ± 0.57	4.25 ± 1.13	4.52 ± 1.13	3.99 ± 0.69
Control muscle	4.89 ± 0.81	3.55 ± 0.63	3.99 ± 0.89	3.92 ± 0.36
Infected muscle /blood	0.79	0.75	0.89	1.31
Infected muscle /control muscle	1.05	1.20	1.13	1.02

%ID/g indicates percent injected dose per gram of tissue.

## Data Availability

The authors confirm that the data supporting the findings of this study are available within the article.
